# Efficacy of *Aloe vera*, *Ananas comosus*, and *Sansevieria masoniana* Cream on the Skin Wound Infected with MRSA

**DOI:** 10.1155/2018/4670569

**Published:** 2018-04-19

**Authors:** Yos Adi Prakoso, Chylen Setiyo Rini, Roeswandono Wirjaatmadja

**Affiliations:** ^1^Integrated Laboratory, Faculty of Health, University of Muhammadiyah Sidoarjo, East Java, Indonesia; ^2^Faculty of Veterinary Medicine, University of Wijaya Kusuma Surabaya, East Java, Indonesia

## Abstract

The tropical area has a lot of herbal medicines such as *Aloe vera* (AV), *Ananas comosus* (AC), and *Sansevieria masoniana* (SM). All the three have a unique potential effect as an antibacterial and wound-healing promoter. The aim of this study is to explore the role of AV, AC, and SM on the skin wound infected with methicillin-resistant *Staphylococcus aureus* (MRSA). Forty-five adult female Sprague Dawley rats weighing 250–300 grams were divided into 5 groups. All the groups were exposed to two round full-thickness punch biopsy and infected with MRSA. The group C was the control group/untreated; group BC was treated with base cream/without extract; group AV was treated with 75% AV cream; group AC was treated with 75% AC cream, and group SM was treated with 75% SM cream. The wounds were observed on days 5, 10, and 15. The healing of skin wounds was measured by a percentage of closure, skin tensile strength, and histopathology. The result showed that AV, AC, and SM have a similar potential effect on healing in the wound that was infected with MRSA compared to the groups C and BC (*P* < 0.05). It shows that all the three herbal formulations can be used as the alternative therapy to the wound infected with MRSA.

## 1. Introduction

*Staphylococcus aureus* is a common opportunistic bacterium in both human skin and animal skin [1]. This bacterium became a serious problem as it is resistant to methicillin. Nowadays, methicillin-resistant *S. aureus* (MRSA) is a worldwide problem and is not limited geographically [2]. It is also one of the leading bacteria causing skin and soft tissue infection [3]. MRSA infection causes significant morbidity and mortality [4], including septic shock, endocarditis, pneumonia, and bacteremia [5]. The major route of infection of MRSA is via open wounds [6]. Open wounds such as excisional wound are complicated by infection, and they cause prolonged and delayed wound healing [7]. The delayed wound healing may be because there is a failure of CD8+ in another mediated immune response to eliminate the bacteria [8]. The failure is caused by incorrect treatments or high bacterial concentration [9].

Herbal medicine is the alternative therapy to promote wound healing by activating the immunological complex [10]. It is reported that the active ingredients of *Aloe vera* (AV) could be used as antibacterials and wound-healing promoters [11]. On the other hand, *Ananas comosus* (AC) contains bromelain, and it is widely administered as an anti-inflammatory and wound-healing agent [12]. The previous study reported the medicinal effects of another *Sansevieria* sp. [13, 14]; however, the efficacy of *Sansevieria masoniana* (SM) in wound healing is still complicated. The aim of this study is to explore the efficacy of AV, AC, and SM on skin wound infected with MRSA.

## 2. Materials and Methods

### 2.1. Methicillin-Resistant S. aureus (MRSA) Isolate

The isolate of MRSA was obtained from the Department of Microbiology, Faculty of Veterinary Medicine, University of Gadjah Mada, Yogyakarta, Indonesia. The biochemical and resistance stability of MRSA was reconfirmed by a standard laboratory test and cefoxitin (30 *µ*g) disc diffusion method before use in this study [15]. All the experimental procedures were conducted in the Integrated Laboratory, Faculty of Health, University of Muhammadiyah Sidoarjo from April until November 2017.

### 2.2. Preparation of Extracts

The plant materials (AV, AC, and SM) were obtained from Batu, Malang. The plants were dried and extracted using ethanol in the Soxhlet extractor. The extract was filtered using Whatman filter paper number 1 under reducing atmospheric pressure. The extract was stored at −20°C before use. The extracts were dissolved in 5% dimethyl sulfoxide (DMSO) for antimicrobial susceptibility tests.

### 2.3. Qualitative Phytochemical Screening

The qualitative phytochemical screening of AV, AC, and SM extract was undertaken using standard methods. The extracts were screened for several components such as tannin, saponin, flavonoid, alkaloid, phenol, and glycoside.

### 2.4. Antimicrobial Test

The MRSA isolate was tested for antimicrobial susceptibility using the disc diffusion method on the Mueller–Hinton agar (MHA). The MRSA was transferred to the nutrient broth and incubated (6 hours) until it showed a turbidity of 0.5 McFarland standard. The suspension was spread on the MHA using sterile cotton swabs. The blank discs (6 mm, Oxoid, CT0998B) were impregnated with 0%, 25%, 50%, 75%, and 100% concentration of the plant extracts which were dissolved in DMSO. The plates were incubated at 37°C for 24 hours. The complete inhibition zone was measured after 24 hours.

Furthermore, the complete inhibition zone was analyzed to determine the percentage inhibition of diameter growth (PIDG) according to the equation below [16]:

(1) $$\begin{array}{c} \text{PIDG}\;\;\left(\%\right)=\frac{A-B}{B}\times 100, \end{array}$$

where *A* is the diameter of inhibition of the extract and *B* is the diameter of inhibition of the solvent.

### 2.5. Cream Formulation

The effective concentration of plant extracts that showed intermediate and/or susceptible results in the antimicrobial test were prepared into cream-based formulation. The cream was prepared with the mixture of oil and water phases. The oil phase (stearic acid, potassium hydroxide, and glycerin) was melted at 70°C and constantly stirred. At the same temperature, the water phase (methylparaben, propylparaben, water, and plant extracts) was prepared. The oil phase was added to the water phase slowly with constant stirring. The cream was prepared daily.

### 2.6. Experimental Animals

This study used forty-five adult female Sprague Dawley rats weighing 250–300 grams provided by the commercial breeder. The rats were placed inside the acrylic aquarium individually and adapted for at least 7 days under the standard laboratory condition (12/12 hours light/dark, standard laboratory animal feed, and water ad libitum). The approval for all animal protocols was obtained from the ethical clearance committee of the University of Gadjah Mada, Yogyakarta, Indonesia, and all the procedures followed the federal guidelines for the care and use of laboratory animals.

### 2.7. Experimental Procedure and Design

The rats were divided into 5 groups. The rats were shaved and anaesthetized using ketamine 50 mg/kg BW and xylazine 4 mg/kg BW. 70% isopropyl alcohol was swabbed on the skin. The skin was exposed to two round 4 mm full-thickness punch biopsy using a sterile disposable punch tool. Each wound was covered with the sterilized transparent dressing and inoculated with 10^5^ colony forming units (CFU) of MRSA in 30 *µ*L of phosphate buffer saline (PBS).

Group C was the control group/untreated; group BC was treated with base cream/without extract; group AV was treated with 75% AV cream; group AC was treated with 75% AC cream; and group SM was treated with 75% SM cream. The treatments were given twice a day at 6/6 (am/pm) for 15 days, and it was started on day one after wounding. The treatments were applied topically using sterile cotton swabs.

### 2.8. Wound Measurement

The wound of each group was recorded and measured as a percentage closure on days 5, 10, and 15. The wound tensile strength was measured using tensometer on day 15 under anaesthesia. The tensile strength was recorded in grams.

### 2.9. Histopathology and Immunohistochemistry

On days 5, 10, and 15, three rats from each group were euthanized using the combination of ketamine and xylazine lethal doses. The skin wound from each group was stored in 10% neutral buffer formalin for 24 hours and processed with standard laboratory techniques for histopathology using hematoxylin and eosin (H&E) staining and collagen deposition using Mallory staining.

For immunohistochemistry, the tissue sections were mounted on the slides coated with polylysine. The sections were stained with monoclonal antibody for CD8+ (anti-rat CD8+, Novocastra, RTU-CD8-295, Cat. number PA0183) using the standard staining protocol of this product.

### 2.10. Morphometry

The slides were randomly analyzed by 2 different pathologists under blindfold condition. The semiquantitative assessment using a scoring system 0–3 (absence (0); mild (1); moderate (2); marked (3)) for each section was performed on the angiogenesis, inflammatory cell infiltration, fibroblast, collagen deposition, and the expression of CD8+ [17].

### 2.11. Analysis Data

The data were analyzed by SPSS 16 and were presented as mean ± standard deviation (SD). The tensile strength and percentage of wound closure were analyzed with ANOVA and post hoc test; however, the histopathological assessment results were analyzed with the Kruskal–Wallis test and Mann–Whitney *U* test.

## 3. Results

In the preliminary study of the qualitative phytochemical screening of all the three extracts showed various components such as tannin, saponin, flavonoid, alkaloid, phenol, and glycoside. However, all the three have tannin, saponin, and alkaloid (Table 1). In addition, the antimicrobial activity of AV, AC, and SM on the MRSA isolate showed similar results based on the disc diffusion test. The 75% concentration of the plant extracts was effective against the MRSA isolate, and it was proved by the complete inhibition zone in intermediate results for AC and SM extracts and, however, susceptible results for the AV extract (Table 2).

**Figure 1:**
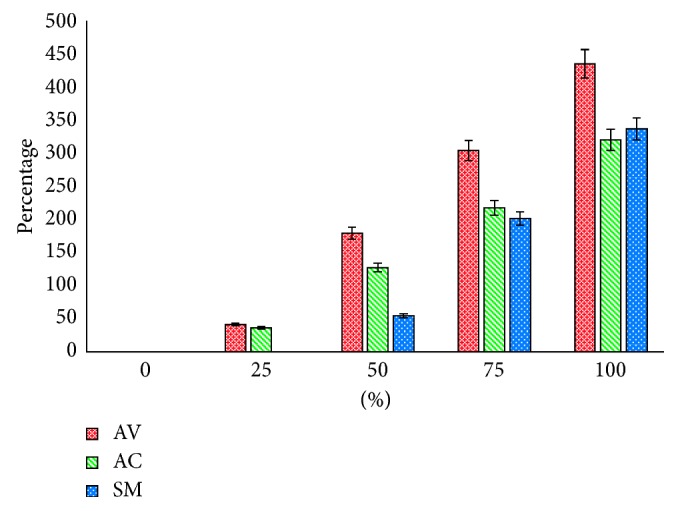
The PIDG indicates the percentage inhibition of MRSA against some concentration of AV, AC, and SM extracts.

Based on the determination of PIDG, AV showed the higher inhibition ability compared with AC and SM (Figure 1). However, all the three extracts have potential effects against MRSA in vitro.

Groups C and BC did not show any differences in all parameters (*P* > 0.05). These results showed that the base cream formulation in this study has no any potential effect on the wound healing. The treatment groups (AV, AC, and SM) showed the better result of healing on the wound that was infected with MRSA in all parameters compared with groups C and BC (*P* < 0.05). Topical application of AV, AC, and SM increased the wound contraction, skin tensile strength, angiogenesis, fibroblast, and collagen deposition in wound tissue, and it starts on day 5 (*P* < 0.05) (Table 3).

**Figure 2:**
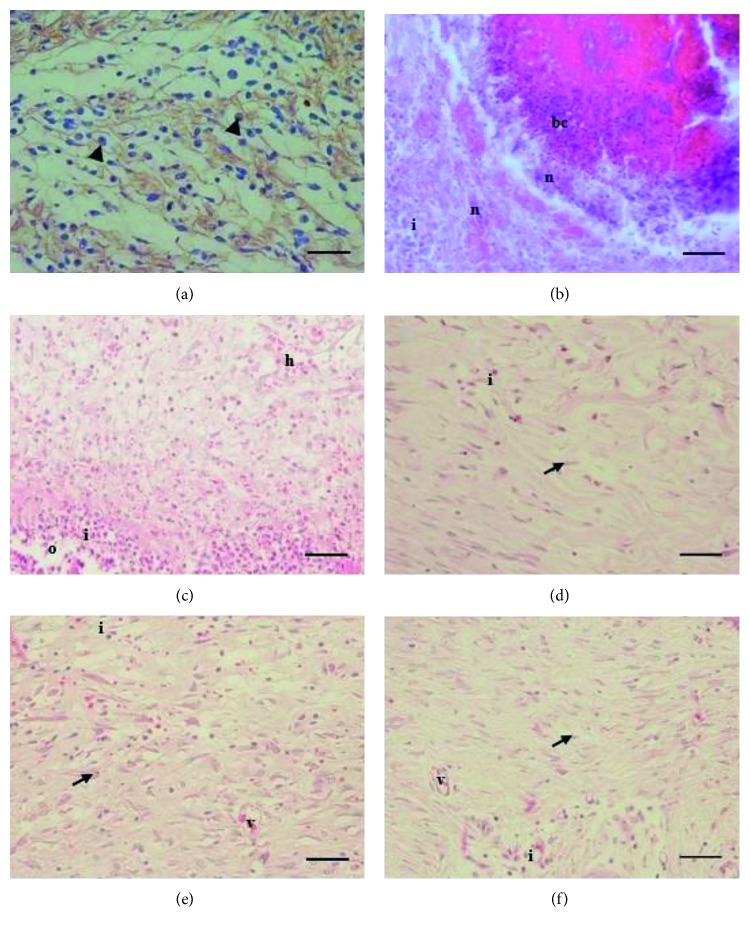
Photomicrographs of the dermal part of the skin wound healing. Predominantly expression of CD8+ on day 5 in treatment group (a); skin wound of control group on day 5 showed severe necrosis with bacterial colonization (b); dermis of group BC (c); group AV (d); group AC (e); and dermis of group SM on day 15 (f). Note: CD8+ expression (arrowhead); fibroblast (arrow); bacterial colonization (bc); haemorrhage (h); inflammatory cells infiltration (i); necrosis (n); edema (o); neovascularization (v). CD8+, DAB, 100x, scale bar: 50 *µ*m (a); H&E, 400x; scale bar: 50 *µ*m (b–f).

In addition, AV, AC, and SM application decreased the inflammatory cell infiltration along with the wound healing (*P* < 0.05). In this study, the better healing of an infected wound is supported by infiltration of CD8+ in the wound tissue. The treatment groups showed the predominant infiltration of CD8+ on day 5 (Figure 2(a)) and sequentially decreased on days 10 and 15 (*P* < 0.05) (Table 3). Surprisingly, the bacterial colonization was shown in the control group on day 5 (Figure 2(b)) with mild infiltration of CD8+.

**Figure 3:**
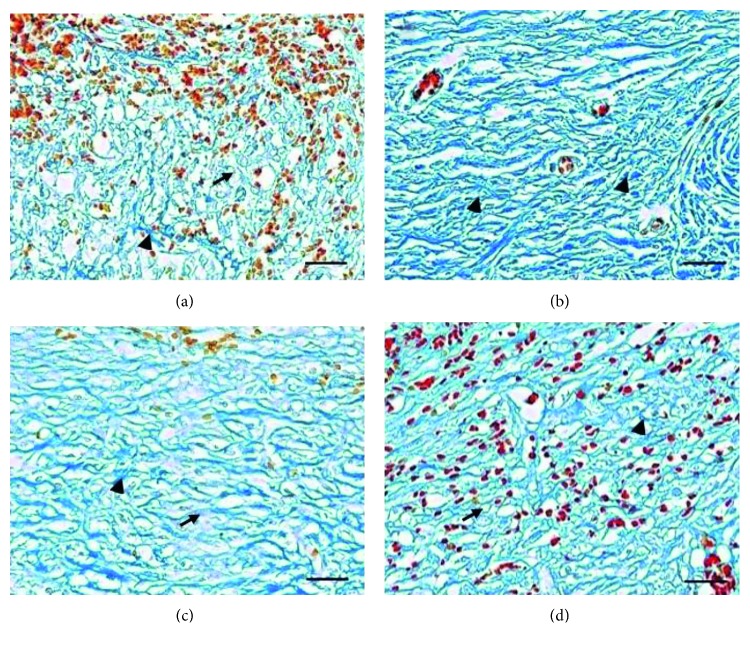
Photomicrographs of collagen deposition in the dermal part of the skin wound healing on day 15. Collagen deposition of group BC with predominant haemorrhage (a); predominantly old collagen deposition in the dermal part of group AV (b); collagen deposition of group AC (c); group SM (d). Note: erythrocytes (orange-red); new collagen fiber (light blue/arrow); old collagen fiber (deep blue/arrow head). Mallory, 400x; scale bar: 50 *µ*m (a–d).

Topical application of AV, AC, and SM has a similar potential effect on angiogenesis, inflammatory cells infiltration, and fibroblast (Figure 2(c)–2(f)); collagen deposition (Figure 3(a)–3(d)); and CD8+ compared with groups C and BC. However, AV and AC have better effects on the percentage of wound closure and skin tensile strength compared with SM (*P* < 0.05) (Table 3). Both groups C and BC showed the weak skin tensile strength (Table 3), and it was supported by the minimal expression of collagen deposition in the dermal part with severe haemorrhage on day 15 (Figure 3(a)). On the other hand, the better collagen deposition appeared along with the skin tensile strength in the dermal part of the herbal treatment groups (Table 3; Figure 3(b)–3(d)). It proved that collagen deposition has a significant correlation with the skin tensile strength.

## 4. Discussion

AV, AC, and SM have been used as traditional medicines containing various components. All the three herbs have different components: AV (tannin, saponin, flavonoid, alkaloid, and glycoside); AC (tannin, saponin, flavonoid, and alkaloid); and SM (tannin, saponin, alkaloid, phenol, and glycoside) (Table 1). Those various components are secondary metabolites produced by plants to fight against microorganisms in the environment [18], and they can be used as herbal antibacterial agents in vitro [19–21].

Both tannin and alkaloid have a potential effect against several pathogens such as bacteria [22, 23], and they could be used as anti-inflammatory agents [24] and also in herbal treatment. However, saponin itself can be used as the anti-inflammatory agent [25]. Based on this study, the extracts have similar effects in inhibiting MRSA in vitro. It is caused by tannin and alkaloid properties inside the AV, AC, and SM extracts. Tannin and alkaloid are known as the major active compounds against MRSA [26].

The advanced wound care strategy is needed to prevent MRSA invasion via open wounds by inhibiting the bacterial colonization and by triggering progressively T-cell subset infiltration in the wound area. One of the T-cell subsets which play an important role in eliminating the infected area is CD8+ [27]. This study proved that AV, AC, and SM are widely used as traditional medicine and have a potential role in activating CD8+ to infiltrate the wound tissue as the mechanism to eliminate MRSA infection [28]. As T-cells are cytotoxic, CD8+ plays an important role not only in controlling the infection but also in eliminating the infected cells [29].

The bacterial colonization in the wound is inhibited by the antimicrobial properties of AV [30], AC [31, 32], and SM, which support the function of CD8+ in wound healing and show the synergic effect when compared with groups C and BC. In addition, the inhibition of bacterial colonization may shorten the inflammation period via cyclooxygenase and prostaglandin route [33]. At the end of an inflammatory phase, both CD4+ and CD8+ release various cytokines to stimulate cell migration, proliferation, and deposition of extracellular matrix [34]. The cell migration is facilitated by angiogenesis or new vessels in the wound tissue. Angiogenesis facilitates fibroblast, keratinocyte, inflammatory cells, and various types of the cytokines to infiltrate the wound tissue [35]. This study proved that fibroblasts synthesize the collagen to support the tissue matrix and collagen deposition supports the skin tensile strength [36]. It is similar to the result of this study that showed the better angiogenesis formation, fibroblast infiltration, collagen deposition, and skin tensile strength in the herbal treatment groups.

## 5. Conclusions

The present study demonstrated that *Aloe vera* (AV), *Ananas comosus* (AC), and *Sansevieria masoniana* (SM) have potential effects to promote the healing of infected wounds.

## Figures and Tables

**Table 1:** Qualitative phytochemical analysis of AV, AC, and SM extracts.

Variable	Extract		
	AV	AC	SM
Tannin	+	+	+
Saponin	+	+	+
Flavonoid	+	+	—
Alkaloid	+	+	+
Phenol	—	—	+
Glycoside	+	—	+

+ = present; — = absent.

**Table 2:** Complete inhibition zone (mm) of the plant extracts in several concentrations.

Extract	Concentration				
	0%	25%	50%	75%	100%
AV	—	8.46 ± 0.30	16.76 ± 0.61	24.23 ± 0.20^∗^	32.10 ± 0.20
AC	—	8.16 ± 0.41	13.63 ± 0.20	19.06 ± 0.20^∗^	25.20 ± 0.26
SM	—	—	9.26 ± 0.55	18.10 ± 1.60^∗^	26.20 ± 0.45

^∗^The effective concentration results based on disc diffusion method; — = absent.

**Table 3:** Effects of the herbal cream formulation on the skin wound infected with MRSA.

Parameter	Group	Day		
		5	10	15
Percentage of wound closure (%)	1 (C)	3.60 ± 1.05	29.94 ± 2.72	54.36 ± 5.75
	2 (BC)	4.51 ± 1.37	32.04 ± 3.88	59.63 ± 2.69
	3 (AV)	10.00 ± 0.76^∗∗^	64.17 ± 4.86^∗∗^	99.10 ± 1.18^∗∗^
	4 (AC)	12.82 ± 4.07^∗∗^	63.67 ± 5.80^∗∗^	99.06 ± 1.62^∗∗^
	5 (SM)	9.25 ± 0.78^∗^	42.04 ± 9.13^∗^	87.02 ± 9.49^∗^
Skin tensile strength (grams)	1 (C)	NT	NT	218.33 ± 9.50
	2 (BC)	NT	NT	219.33 ± 5.85
	3 (AV)	NT	NT	578.00 ± 12.00^∗∗^
	4 (AC)	NT	NT	566.67 ± 28.29^∗∗^
	5 (SM)	NT	NT	500.67 ± 15.56^∗^
Angiogenesis	1 (C)	0.50 ± 0.54	0.83 ± 0.40	1.00 ± 0.63
	2 (BC)	0.50 ± 0.54	1.33 ± 0.51	1.66 ± 0.51
	3 (AV)	1.50 ± 0.54^∗^	2.16 ± 0.40^∗^	2.66 ± 0.51^∗^
	4 (AC)	1.33 ± 0.51^∗^	1.83 ± 0.75^∗^	2.66 ± 0.81^∗^
	5 (SM)	1.16 ± 0.75^∗^	1.83 ± 0.75^∗^	2.50 ± 0.83^∗^
Inflammatory cells infiltration	1 (C)	3.00 ± 0.00	2.00 ± 0.89	2.00 ± 0.63
	2 (BC)	3.00 ± 0.00	2.66 ± 0.51	2.16 ± 0.75
	3 (AV)	2.50 ± 0.54^∗^	1.83 ± 0.75^∗^	0.83 ± 0.75^∗^
	4 (AC)	2.33 ± 0.51^∗^	2.00 ± 0.63^∗^	1.00 ± 0.63^∗^
	5 (SM)	2.50 ± 0.83^∗^	1.50 ± 0.83^∗^	1.33 ± 0.51^∗^
Fibroblast	1 (C)	0.66 ± 0.51	1.16 ± 0.75	1.50 ± 0.54
	2 (BC)	0.66 ± 0.81	1.50 ± 0.54	1.50 ± 0.54
	3 (AV)	1.66 ± 0.51^∗^	2.00 ± 0.63^∗^	2.50 ± 0.83^∗^
	4 (AC)	1.33 ± 0.81^∗^	1.83 ± 0.40^∗^	2.66 ± 0.51^∗^
	5 (SM)	1.33 ± 0.81^∗^	2.00 ± 0.63^∗^	2.66 ± 0.51^∗^
Collagen deposition	1 (C)	0.16 ± 0.40	0.83 ± 0.40	1.33 ± 0.81
	2 (BC)	0.33 ± 0.51	1.00 ± 0.63	1.33 ± 0.81
	3 (AV)	1.16 ± 0.40^∗^	1.66 ± 0.51^∗^	3.00 ± 0.00^∗^
	4 (AC)	0.66 ± 0.51^∗^	1.66 ± 0.51^∗^	2.50 ± 0.54^∗^
	5 (SM)	1.00 ± 0.63^∗^	1.66 ± 0.51^∗^	1.66 ± 0.51^∗^
Expression of CD8+ lymphocytes	1 (C)	0.16 ± 0.40	0.83 ± 0.75	1.33 ± 0.81
	2 (BC)	0.33 ± 0.51	0.66 ± 0.51	1.50 ± 0.54
	3 (AV)	2.83 ± 0.40^∗^	2.33 ± 0.51^∗^	0.66 ± 0.51^∗^
	4 (AC)	2.33 ± 0.81^∗^	2.16 ± 0.75^∗^	1.00 ± 0.63^∗^
	5 (SM)	2.00 ± 1.26^∗^	2.00 ± 0.63^∗^	1.50 ± 0.54^∗^

NT, not tested; the values are expressed as mean ± SD; ^∗^/^∗∗^the different superscript on the same column and parameter showed significantly different values (*P* < 0.05).
